# Interaction between Depth Order and Density Affects Vection and Postural Sway

**DOI:** 10.1371/journal.pone.0144034

**Published:** 2015-12-02

**Authors:** Astrid J. A. Lubeck, Jelte E. Bos, John F. Stins

**Affiliations:** 1 Department of Human Movement Sciences, Faculty of Behavioural and Movement Sciences, Vrije Universiteit Amsterdam, MOVE Research Institute Amsterdam, Amsterdam, The Netherlands; 2 TNO Perceptual and Cognitive Systems, Soesterberg, The Netherlands; University of Akron, UNITED STATES

## Abstract

**Objective:**

Vection, a feeling of self-motion while being physically stationary, and postural sway can be modulated by various visual factors. Moreover, vection and postural sway are often found to be closely related when modulated by such visual factors, suggesting a common neural mechanism. One well-known visual factor is the depth order of the stimulus. The density, i.e. number of objects per unit area, is proposed to interact with the depth order in the modulation of vection and postural sway, which has only been studied to a limited degree.

**Methods:**

We therefore exposed 17 participants to 18 different stimuli containing a stationary pattern and a pattern rotating around the naso-occipital axis. The density of both patterns was varied between 10 and 90%; the densities combined always added up to 100%. The rotating pattern occluded or was occluded by the stationary pattern, suggesting foreground or background motion, respectively. During pattern rotation participants reported vection by pressing a button, and postural sway was recorded using a force plate.

**Results:**

Participants always reported more vection and swayed significantly more when rotation was perceived in the background and when the rotating pattern increased in density. As hypothesized, we found that the perceived depth order interacted with pattern density. A pattern rotating in the background with a density between 60 and 80% caused significantly more vection and postural sway than when it was perceived to rotate in the foreground.

**Conclusions:**

The findings suggest that the ratio between fore- and background pattern densities is an important factor in the interaction with the depth order, and it is not the density of rotating pattern *per se*. Moreover, the observation that vection and postural sway were modulated in a similar way points towards a common neural origin regulating both variables.

## Introduction

Seeing visual motion while being physically stationary, can cause significant perceptual- and motor effects in humans. A well-known effect is a feeling of self-motion, called vection [1]. Also postural control [2], is known to be affected by visual motion [3–5].

Vection can be evoked by viewing linear as well as circular motion, coined linear vection and circular vection, respectively. Circular vection caused by stimuli rotating around an Earth-vertical axis is known to be saturated [1,6], i.e., a sensation of self-motion completely opposite to the motion direction of the stimulus, with the stimulus being perceived as stationary. In this study, however, we focus on the effects of viewing roll-motion around the naso-occipital axis. This type of visual roll-motion is known to lead to a tilt of the perceived orientation of the Earth-vertical [7–9], which is arguably caused by the discrepancy between visual, vestibular, and proprioceptive information with respect to the Earth-vertical (see e.g. [1,9–11]). The perceived tilt of the Earth-vertical is known to lead to a sensation of static self-tilt, here defined as roll-vection [1].

Well-known parameters related to vection are its latency and its duration, known to be influenced by various visual factors [12–15]. Such visual factors can be useful in optimizing user experience in virtual environments, see for example [12,13,16]. An important visual factor is the perceived depth order of the motion stimulus [6,12,13,17–20]. In experiments, typically, two patterns–one stationary and one rotating–are presented simultaneously within the same viewing area. By virtue of certain visual cues, such as occlusion or stereoscopic depth, the viewer will perceive one pattern as being in front of the other pattern. When a moving pattern is perceived behind a stationary pattern vection is facilitated, whereas a moving pattern perceived in front of stationary pattern typically reduces vection [14,17,18,21]. Seno et al. [22] extended this so called fore-background hypothesis with the object-background hypothesis, which states that vection is primarily induced by the perceived moving background, while a pattern perceived as an object in motion will hardly be effective in inducing vection.

Intuitively, such a fore-background or object-background effect would suggest that other visual factors related to the perceived foreground and background can differentially influence the effects of the depth order. Factors such as stimulus size, eccentricity and density of the stimulus, indeed have shown to interact with the perceived depth order in the modulation of vection [14,15,17,23]. Here, density indicates *number density*, i.e., the number of particles or objects per unit area. Moreover, we confined ourselves to the term *density*, implying the relative number of objects with respect to a chosen maximum (see Methods).

Already in 1975 Brandt et al.[17] studied the interaction between the perceived depth order and density on roll-vection. In one experiment the density of a stationary pattern, perceived behind a pattern in roll, was varied to study its effect on roll-vection. Results revealed that an increase in density of this stationary background pattern significantly decreased roll-vection duration. In a second experiment it was demonstrated that increasing the density of a rotating pattern, shown in the absence of a stationary pattern, caused an increase in roll-vection duration [17].

With this study Brandt et al.[17] were the first to show that the perceived depth order interacted with density in the modulation of roll-vection. Although this study provided a significant stepping stone for more research, to date no study yet has systematically studied the interaction between the perceived depth order and density of both patterns. With the currently described study we aim to fill this gap.

Besides vection, it is well-known that also postural sway can be influenced by visual motion [3–5,24]. When exposed to a pattern rotating around the naso-occipital axis or a horizontal translating pattern, body posture typically deviates in the direction of the visual motion. That is, with counter clockwise or leftward motion and clockwise or rightward motion, postural sway deviates leftward or rightward, respectively [1,5,7,24]. Similar to roll-vection, these postural deviations are thought to reflect the perceptual bias of the Earth-vertical [11] caused by exposure to roll-motion. Moreover, several studies have proposed that lateral postural deviations and vection are closely related, and are mediated through a common neural mechanism [5,24,25]. A first finding substantiating this hypothesis is that when an observer experiences vection, postural sway deviates further into the direction of stimulus motion compared to episodes without vection [5,24,25]. Second, just as vection, postural sway can be modulated by visual factors such as eccentricity, temporal frequency [26] and also density [3] of the visual pattern. Lestienne [3], for example, showed that a moving pattern with a higher density caused larger postural excursions. But also for postural sway, it has not yet been studied systematically whether it is affected by the interaction between the perceived depth order and density. If postural sway and roll-vection are modulated in a similar way, it would provide additional evidence for a common neural origin.

To investigate the above addressed aims we performed an experiment in which the densities of a stationary and a rotating pattern were systematically varied, while the perceived depth order (rotating pattern occluding or being occluded by the stationary pattern) was also varied. Based on earlier findings we hypothesize to find an interaction between perceived depth order and density in the modulation of roll-vection and postural sway. Moreover, since it is postulated [5,24,25] that vection and postural sway share a common neural origin, we expect postural sway to be affected in a similar way as roll-vection by the perceived depth order, density and the interaction.

## Method

### Participants

Seventeen adults participated voluntarily after signing an informed consent form. Participants were students and members of the Faculty of Human Movement Sciences of the VU University, 6 males and 11 females with a mean age of 26.7 years (*SD* = 4.3 years). All participants were free of a history of ocular and vestibular disorders. Ethical approval in accordance with the Declaration of Helsinki was provided by the Ethics Committee of the same faculty.

### Apparatus and Stimuli

In a completely darkened room, participants were exposed to stimuli shown on a 55 inch (121.8 x 68.5 cm; width x height) TV-screen (LG 55LA8609) in high definition resolution (1920 x 1080 pixels). Participants stood at a distance of 70 cm from the screen, yielding a Field of View (FoV) of approximately 52 x 52°. All possible Earth-fixed cues made visible by scattered light were eliminated using the following precautions. First, participants were asked to wear neutral density glasses passing only 1% of light. Second, the edges of the screen were masked using a low-reflective cardboard cover that left a circular viewing area with a radius of 34 cm visible.

**Fig 1:**
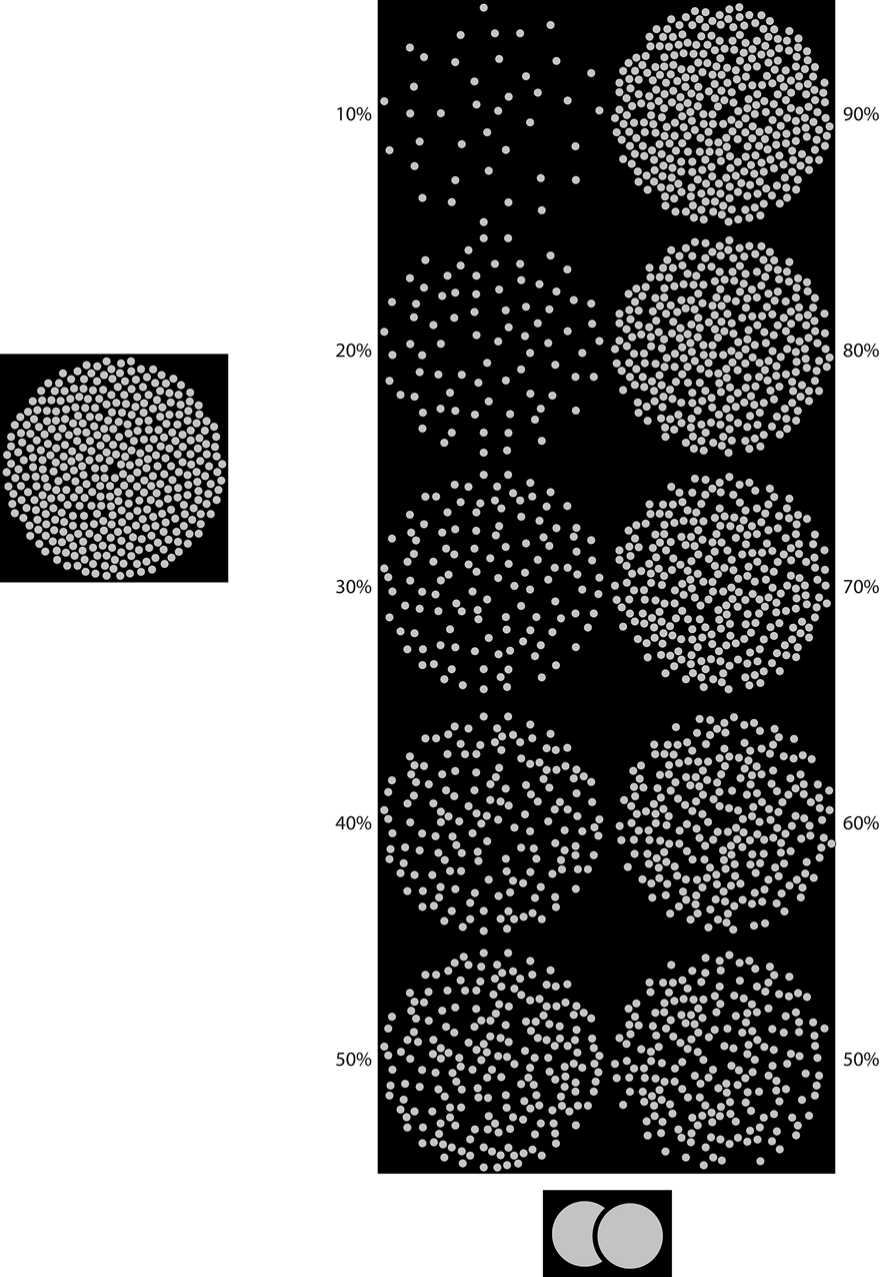
Combinations of patterns used to study the interaction between depth order and pattern densities. The number of dots per pattern, i.e., the pattern density, was varied in steps of 10% between 10 and 90% of the total number of dots. The densities of both patterns combined always added up to 100%. Of each combination, one of the patterns rotated in roll, in front of or behind the other that remained stationary. The inset shows a dot occluding another dot so that the black (outside) border became visible. The black borders of the dots were clearly visible when one of the patterns was rotating with respect to the other, since the background was black as well.

Stimuli consisted of two dot patterns that together composed one stimulus (Fig 1; S1 and S2 Videos). The two patterns combined always contained 400 dots. The number of dots per pattern, i.e., the density of each pattern, was varied in steps of 10% between 10 and 90% of the total number of dots. The densities of both patterns combined always added up to 100%. The dots were positioned quasi randomly, such that within each pattern they did not overlap or touch, each dot subtending a FoV of approximately 2°. All dots were surrounded by black borders that were only visible when one of the patterns was rotating with respect to the other, since the background displayed behind both patterns was black as well (inset Fig 1; S1 and S2 Videos). During rotation one pattern appeared to rotate in front of the other due to occlusion. In other words, the occluding pattern (foreground) was rotating while the occluded pattern (background) was stationary, or vice versa. Combinations of the depth order (foreground or background rotation) and the different densities of the patterns resulted in a total of 18 trials; 9 trials for each plane of rotation with the density of the rotating pattern ranging from 10 to 90% in steps of 10%. The rotating pattern had a velocity of 30°/s and rotated either clockwise or counter clockwise as explained in the procedure. This design enabled us to study the interaction between perceived depth order and density.

During exposure, participants were standing barefoot on a custom made 1 x 1 m strain gauge force plate, recording the Centre of Pressure (CoP) at 100 Hz. Participants stood with a computer mouse in both hands, used to obtain data on roll-vection, and had their feet positioned with the heels together with a 30° angle between the feet as marked on the force plate. The start of exposure to the stimulus was synchronized with the start of the CoP measurement by means of a light sensor that recorded when the stimulus appeared on the screen.

### Procedure

At the beginning of the experiment, the procedure was explained to the participants, followed by signing the informed consent form. Next, participants were asked to stand on the force plate as instructed, and to put on the neutral density glasses. Participants were instructed to look at the centre of the stimulus, to stand as quietly as possible, and to only take a step to prevent falling (which never happened). Finally, participants held a computer mouse in both hands in front of their body and were asked to press a button when they experienced roll-vection, i.e. a sensation of self-motion or self-tilt, and to release the button when they no longer experienced roll-vection. Vection latency was defined as the time interval between rotation onset and the first button press. Vection duration was defined as the total time that participants pressed a button during exposure to the rotating pattern within one trial.

The experiment was divided into two sessions with 9 trials. In the first session the 9 density combinations were presented in a randomized order together with a randomization of the depth order (i.e. foreground or background rotation). In the second session the 9 density combinations were again randomized, but for each density combination the depth order was counter balanced in comparison with the first session. Thus, at the end of the experiment all 18 combinations of depth order and density were viewed by the participant.

Each trial lasted 88 s, and was divided into three uninterrupted phases. The first (pre-)phase lasted 20 s, during which participants were exposed to a stationary stimulus pattern, allowing us to collect baseline CoP data. This pre-phase was followed by a rotation-phase in which one of the dot patterns of the stimulus rotated for 48 s (4 revolutions with a velocity of 30°/s). During this rotation-phase participants pressed a button when they experienced roll-vection. The choice for a rotation-phase of 48 s was a trade-off between the time needed to reliably induce roll-vection, as tested in a pilot experiment, and to keep the duration of the of the experiment within acceptable limits. In the third (post-)phase participants were again exposed to a stationary stimulus for 20 s. This final phase allowed us to collect data on possible postural after-effects of rotation. The rotation direction in the rotation phase (clockwise and counter clockwise) was alternated between trials in order to prevent accumulation effects.

### CoP analysis

First, the CoP time series were filtered with a 4^th^ order low-pass Butterworth filter with a cut-off frequency of 5 Hz. Second, CoP time series obtained during CCW pattern rotation were mirrored for the ML direction. Positive CoP values therefore denote a position shift into the direction of rotation and negative CoP values denote the opposite. From these series we calculated three measures for all phases separately: (1) the sway path length (SPL), (2) the moving window standard deviation (MWSD) in ML direction, and (3) the lean in ML direction. The SPL was defined as the length the CoP travelled during each phase in the AP-ML plane. For the MWSD and the lean only the ML CoP time series were analysed, because rotation in the frontal plane elicited the largest shifts in this direction. For calculation of the MWSD for each phase, SDs of 1 s non-overlapping time windows of 100 data points were computed, which were then averaged to obtain a MWSD. The MWSD was favoured over a standard deviation calculated over the entire phase, because of large, relatively slow changes in CoP position in response to rotation of the pattern [27]. In other words, with this measure we were able to quantify postural sway variability independent of large, relatively slow changes in the CoP position. With the lean, the average positional CoP shift in ML direction during rotation (rotation phase), relative to before rotation (still phase) was quantified.

### Statistical analysis

IBM SPSS Statistics 21 was used for statistical analysis. Vection duration and latency (see S1 Data) were investigated using non-parametric tests. If a participant reported no roll-vection at all, the duration was set at 0 s and the latency was (arbitrarily) set at 48 s for that particular trial. Note that these values do not affect the statistics, because of the use of non-parametric, rank based tests. To study the effect of density of the rotating pattern, Friedman tests were conducted for each plane (foreground, background) separately. To compare foreground rotation with background rotation for each density, separate Wilcoxon Signed rank tests were performed on these same variables.

All postural variables (see S2 Data) appeared to meet the assumption of normality, as checked with Shapiro-Wilk tests and by visual inspection of q-q plots. All postural sway variables obtained in the rotation-phase were statistically investigated with separate 2 (foreground, background) x 9 (density ranging from 10% to 90%) repeated measures (RM) ANOVAs. Significant main effects were followed up by difference contrasts. Significant interaction effects were followed up by separate one-way RM ANOVAs for each plane, and paired t-tests for each density. To control for multiple comparisons a Benjamini-Hochberg correction [28,29] was applied, which controls for the false discovery rate (FDR). We chose to control for the FDR instead of the familywise error rate, because the FDR has more statistical power compared to procedures controlling for familywise error rates, e.g. bonferroni techniques [28,29].

To investigate pre-post effects the SPL, MWSD and lean data obtained in the pre-phase were subtracted from data obtained in the post-phase. These difference scores were also subjected to a 2 x 9 RM ANOVA for each postural sway measure separately. Partial eta-squared (*η*_*p*_^*2*^) was calculated to determine the effect-size and the significance level was set at 0.05.

## Results

### Vection

**Fig 2:**
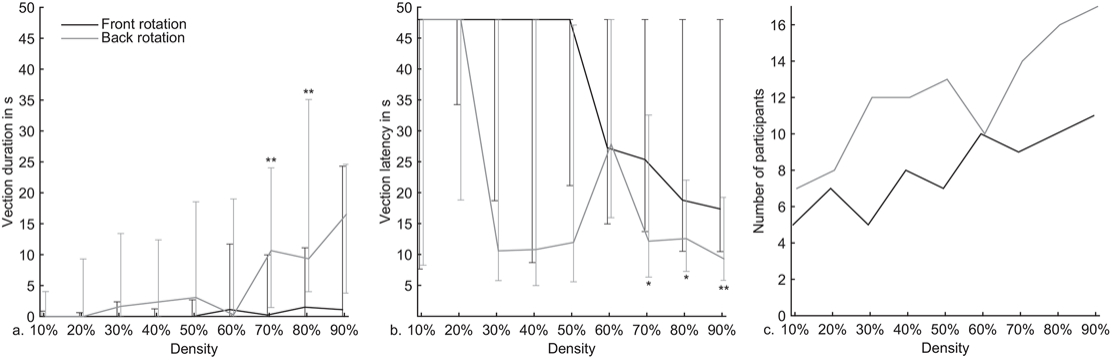
a. Median vection duration, b. median vection latency, and c. the number of participants that reported vection for all combinations of pattern density and perceived depth structure. The error bars represent the 25% and 75% quantiles. Significant differences at *p* < .05 are indicated with *. Significant differences at *p* < .01 are indicated with **.

A larger density of the rotating pattern in the fore- and background significantly increased vection duration, χ^2^(8) = 25.26, *p* = .001 and χ^2^(8) = 39.25, *p* = .000004 respectively. Comparison of foreground rotation with background rotation for each density revealed that at a density 70% and 80% background rotation caused a significantly longer vection duration than foreground rotation, *Z* = 2.95, *p* = .003 and *Z* = 3.35, *p* = .001 respectively (Fig 2A). Vection latency showed similar results, but less pronounced. An increase in density of the rotating pattern did decrease vection latencies with foreground rotation, but failed to reach significance, χ^2^(8) = 13.97, *p* = .083. Only with background rotation a larger density caused the vection latency to decrease significantly, χ^2^(8) = 24.89, *p* = .002. Comparison between foreground and background rotation for each density revealed that a pattern density of 70%, 80% and 90% caused a significantly shorter vection latency when it rotated in the background compared to the foreground, *Z* = 2.27, *p* = .023, *Z* = 2.34, *p* = .019 and *Z* = 2.63, *p* = .009 respectively, (Fig 2B). Fig 2C finally shows that the significant effects in duration and latency were caused by an increasing number of participants that reported vection with an increase in density. Moreover, in line with the statistics, this figure reveals that a greater number of participants reported vection with rotation in the background compared to rotation in the foreground.

### Postural Sway

**Fig 3:**
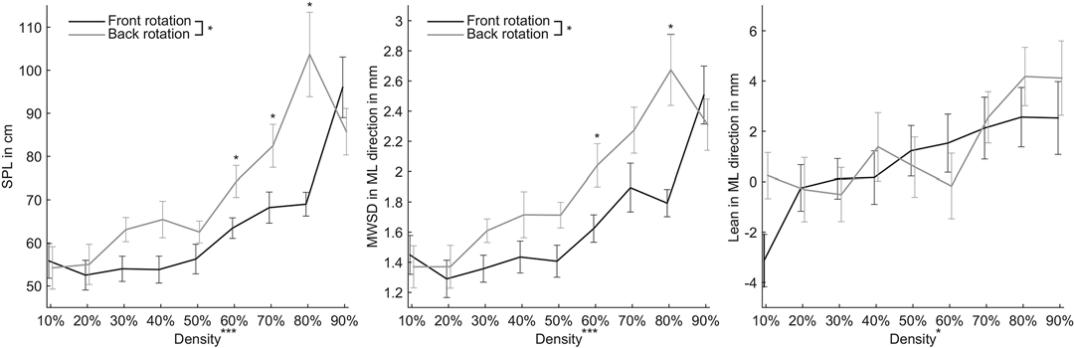
Mean (±SE) sway path length (SPL, left), moving window standard deviation (MWSD, middle) and lean (right) for all combinations of density and perceived depth structure. Significant differences at *p* < .05 are indicated with *. Significant differences at *p* < .01 are indicated with **. Significant differences at *p* < .001 are indicated with ***.

All postural sway measures–the SPL, MWSD and the lean–showed significant main and interaction effects due to variation in depth order and density (Fig 3). For the SPL and MWSD the assumption of sphericity was violated (*p* < .0001), therefore Greenhouse-Geisser corrections were applied. The SPL was affected by both the plane of rotation and the density of the rotating pattern, *F*(1,16) = 11.07, *p* = 0.004, *η*_*p*_^*2*^ = 0.41 and *F*(2.31,36.99) = 13.47, *p* < 0.0001, *η*_*p*_^*2*^ = 0.46 respectively. When the rotating pattern was perceived in the background it caused a larger SPL than when perceived in the foreground. Also, the higher the density of the rotating pattern, the more participants swayed. Statistics also revealed an interaction between depth order and pattern density, *F*(3.07,49.08) = 4.13, *p* = 0.01, *η*_*p*_^*2*^ = 0.21. Investigated with one-way ANOVAs, a greater pattern density caused larger SPLs for both foreground- and background rotation. However, paired t-tests showed that a rotating pattern in the background with a density of 60 and 80% caused significantly larger SPLs than rotation in the foreground (S1 Table). Analysis of the pre-post SPL differences revealed that an overall increase in density caused a significant after-effect, *F*(3.60,57.58) = 3.93, *p* = 0.009, *η*_*p*_^*2*^ = 0.20. Further investigation of this main effect revealed that a rotating pattern with a density of 80% and 90% caused significantly larger after-effects compared to smaller percentages, with both *p* < 0.05.

For the MWSD results comparable to the SPL were found. Background rotation caused a larger MWSD compared to foreground rotation, *F*(1,16) = 12.19, *p* = 0.003, *η*_*p*_^*2*^ = 0.43. And the greater the density of the rotating pattern, the larger the MWSD, *F*(2.87, 48.96) = 12.46, *p* < .0001, *η*_*p*_^*2*^ = 0.44. Again a significant interaction between depth order and density was found, *F*(3.09, 49.38) = 2.81, *p* = .048, *η*_*p*_^*2*^ = 0.15. In the foreground condition, as well as the background condition, a larger density of the rotating pattern caused a larger MWSD, *F*(3.17, 50.64) = 8.45, *p* < 0.0001, *η*_*p*_^*2*^ = 0.35 and *F*(3.83,61.28) = 8.20, *p* < 0.0001, *η*_*p*_^*2*^ = 0.34 respectively. Comparison of the plane of rotation for each density revealed that at a density of 60 and 80% rotation in the background caused a larger MWSD than rotation perceived in the foreground (S1 Table). Also pre-post differences in MWSD showed effects of the density of the rotating pattern, *F*(3.30,52.86) = 3.24, *p* = 0.026, *η*_*p*_^*2*^ = 0.17. Similar to pre-post SPL differences, a rotating pattern with densities of 80% and 90% caused significantly bigger after-effects compared to the lower densities, with both *p* < 0.05.

The lean was only significantly influenced by the density, *F*(4.07, 66.14) = 3.23, *p* = .017, *η*_*p*_^*2*^ = 0.17. Difference contrasts showed that for a pattern in roll with a density of 70% or more, the lean was significantly increased compared to the lower densities. The lean after exposure was not significantly affected by either the plane of rotation or the density.

## Discussion

The main objective of the present study was to investigate the interaction between the perceived depth order and density of the rotating and stationary patterns on roll-vection and postural sway. Moreover, based on the hypothesis that vection and postural sway are modulated via a common neural mechanism, we expected postural sway to be affected in a similar way as roll-vection by the perceived depth order and pattern density, including their interaction.

A first, important finding was that occlusion, the only depth cue in the stimulus, was able to induce a clear effect of depth order on both roll-vection and postural sway. Background roll-motion always resulted in a longer vection duration and shorter latency than foreground rotation. These results are in line with those of previous studies that investigated the effect of depth order on vection using binocular depth cues [5,14,17,18], as well as monocular depth cues [18,30]. Similar to the results for roll-vection, participants also swayed more (SPL, MWSD) when the rotating pattern was occluded by the stationary pattern compared to the opposite configuration. This finding indicates that, similar to roll-vection, postural sway is also modulated by the perceived depth order as realized by occlusion.

Our results also indicate that the density of the rotating pattern plays a significant role in the modulation of roll-vection and postural sway. Irrespective of the plane of rotation, the greater the density of the rotating pattern, the more vection was experienced and the larger postural excursions were. These current results are in agreement with earlier studies that also observed the same modulating effect of density on vection and postural sway [1,3,14,17]. With regard to postural sway, also a significant after-effect in the direction opposite to the stimulus direction was observed, which has also been found in previous studies [1,25,31]. Moreover, also this postural after-effect significantly increased with an increase in density of the rotating pattern.

The most important finding, which is in line with the study of Brandt et al. [17], is that we found the perceived depth order to interact with density in the modulation of roll-vection and postural sway. For roll-vection duration and latency we observed that a rotating pattern with a density of 70% and 80%, combined with a stationary pattern containing respectively 30% and 20% of the dots, caused significantly longer roll-vection durations and shorter latencies when it rotated in the background as compared to the foreground. With respect to postural sway, a rotating pattern with a density of 60 and 80% combined with a stationary pattern containing 40 or 20% of the dots, caused significantly more postural sway when it was perceived to rotate in the background than in the foreground. In other words, the current results indicate that the ratio between the two pattern densities is a factor of importance in the interaction with the perceived depth order, apart from the absolute density of the rotating pattern *per se*.

The conditions containing the rotating pattern with a density of 90% substantiate the above hypothesis. If the absolute density of the rotating pattern would be more important than a ratio in the interaction with depth order, the rotating pattern containing 90% of the dots should have optimized the effect of depth order on roll-vection and postural sway. Moreover, these findings also indicate that the ratio between the density of the rotating and stationary pattern has an optimal range in the interaction with depth order, which can be explained as follows. Initially, an increase of the differential fore-background effect was achieved by increasing the density of the rotating pattern and decreasing the density of the stationary pattern. When approaching a density of 100% for the rotating pattern, and 0% for the stationary pattern, the differential effect would decrease since there is hardly any difference left between fore- or background motion. Thus, a rotating pattern with a density of 90% together with a stationary pattern with a density of 10%, were not able to create a clear fore-background segregation, resulting in no differential effect of the depth order on roll-vection and postural sway.

The current findings on roll-vection and postural sway, are also in agreement with the object-background hypothesis, as proposed by Seno [22]. A pattern with a lower density could be perceived as the object, and a pattern with a higher density could be perceived as the background. The finding that an increasing density of the rotating pattern is accompanied by an increase in roll-vection duration and postural sway, thus substantiates the object-background hypothesis. Yet, the interaction between depth-order and density suggests that a rotating pattern in shown in the foreground needs to have a higher density to be perceived as a background, compared to a similar pattern rotating in the background (i.e. occluded pattern). Summarized, the current findings provide evidence in favour of the object-background hypothesis, but also show that the depth order (induced by occlusion) influences at what density the rotating pattern is perceived as an object or as a background.

An additional explanation for the interaction between density and depth order can be derived from an earlier study performed by Nakamura [14]. He studied the interaction between depth order, stimulus size and eccentricity using a stationary pattern and a horizontal translating pattern using stereoscopic images. The static pattern was either a central dot pattern, subtending 10, 20, 30 or 40°, or a peripheral dot pattern with the central region, that subtended 10, 20, 30 or 40°, left blank. He observed that a larger static central foreground pattern intensified vection, while a larger static peripheral background pattern suppressed vection. Besides the interaction between depth order, eccentricity and stimulus size on vection strength, Nakamura [14] argued that a greater number of stationary elements that were overlapping with the moving pattern when stimulus sizes increased, may have had a significant confounding influence. With the current experiment we have indeed demonstrated that the amount of overlap could have been the confounding factor in his experiment. In our study the combination with the least amount of overlap, did not show an effect of depth order, for which reason it can be concluded that a minimal amount of overlap is needed to promote a differential effect of the perceived depth order. Second, the trials in which both patterns contained 50% of the dots, the combination with the largest amount of overlap, show that it is also not the amount of overlap *per se* that can explain the effect of depth order. If the amount of overlap would be an essential factor, one would expect that this combination maximized the differential effect of depth order on roll-vection and postural sway. Because this was not the case, it is likely that also the amount of overlap within certain limits maximizes the differential effect of depth order on roll-vection and postural sway.

The current findings on the modulation of roll-vection and postural sway also provide an opportunity for further research. In this study we only used only one size of dots and one fixed number of dots, which could be visual factors that can influence the interaction between depth order and density. Future research is needed to unravel to what extent these visual factors modulate the interaction between depth order and density.

A final finding is that postural sway and roll-vection measures were influenced similarly by the perceived depth order, density and their interaction. In line with earlier studies reporting that postural sway and vection were modulated in a similar way [4,5,25,32], these results point towards a common neural origin that modulates vection as well as postural sway. With regard to motion around the naso-occipital axis, a neural representation of verticality could be a working mechanism employed by the central nervous system [9–11]. Several studies have shown that visual roll-motion does influence a neural representation of verticality, and is proposed to regulate Earth-vertical related behaviours, such as postural control [11,33].

Summarized, this study revealed that the density ratio of the rotating pattern to the stationary pattern plays an important role in the interaction with the perceived depth order, modulating both roll-vection and postural sway. In addition, this study has shown that roll-vection and postural sway are modulated in a similar way by the perceived depth order, pattern density and the interaction, possibly reflecting a common neural origin.

## Supporting Information

S1 Data Vection duration data and vection latency data. (SAV)

S2 Data Data on postural sway, including the sway path length, moving window standard deviation and the lean. (SAV)

S1 Table Table showing the paired t-tests following significant interaction effects for the SPL and MWSD. (DOCX)

S1 Video Videos of the stimuli with pattern rotation in the background. (MP4)

S2 Video Videos of the stimuli with pattern rotation in the foreground. (MP4)
