# Supplementary material for: Interaction between Depth Order and Density Affects Vection and Postural Sway
Source: PLoS One. 2015 Dec 2;10(12):e0144034. doi: 10.1371/journal.pone.0144034 (PMC4668089; doi:10.1371/journal.pone.0144034)
Supplement: S1 Table — (DOCX) [file pone.0144034.s003.docx]

Table 1. Paired t-tests following significant interaction effects for the SPL and MWSD.

| **Foreground vs. background** | ***df*** | ***t*** | ***p*** | ***r*** |
| --- | --- | --- | --- | --- |
| **SPL** | | | | |
| 10% | 16 | 0.359 | .724 | .008 |
| 20% | 16 | -0.937 | .363 | .052 |
| 30% | 16 | -1.624 | .124 | .141 |
| 40% | 16 | -2.084 | .053 | .213 |
| 50% | 16 | -1.147 | .268 | .076 |
| 60%** | 16 | -3.606 | .002 | .448 |
| 70% | 16 | -2.149 | .047 | .224 |
| 80%** | 16 | -3.317 | .004 | .407 |
| 90% | 16 | 1.103 | .286 | .071 |
| **MWSD** | | | | |
| 10% | 16 | 0.582 | .569 | .020 |
| 20% | 16 | -0.816 | .427 | .040 |
| 30% | 16 | -1.900 | .059 | .205 |
| 40% | 16 | -1.181 | .255 | .080 |
| 50% | 16 | -1.733 | .102 | .158 |
| 60%** | 16 | -3.026 | .008 | .364 |
| 70% | 16 | -2.030 | .076 | .205 |
| 80%** | 16 | -3.089 | .007 | .374 |
| 90% | 16 | -0.751 | .464 | .034 |

Significant differences at *p* < .05 abd *p* < .01 are indicated with * and **, respectively.
